# Complemented Palindromic Small RNAs First Discovered from SARS Coronavirus

**DOI:** 10.3390/genes9090442

**Published:** 2018-09-05

**Authors:** Chang Liu, Ze Chen, Yue Hu, Haishuo Ji, Deshui Yu, Wenyuan Shen, Siyu Li, Jishou Ruan, Wenjun Bu, Shan Gao

**Affiliations:** 1 Laboratory of Medical Molecular Virology, School of Medicine, Nankai University, Tianjin 300071, China; changliu@nankai.edu.cn (C.L.); huyue2016@mail.nankai.edu.cn (Y.H.); shenwy@mail.nankai.edu.cn (W.S.); 2 State Key Laboratory of Veterinary Etiological Biology and Key Laboratory of Veterinary Parasitology of Gansu Province, Lanzhou Veterinary Research Institute, Chinese Academy of Agricultural Science, Lanzhou 730046, China; chenze@caas.cn; 3 Co-Innovation Center for Prevention and Control of Important Animal Infectious Diseases and Zoonoses, Yangzhou 225009, China; 4 College of Life Sciences, Nankai University, Tianjin 300071, China; haishuo_ji@mail.nankai.edu.cn (H.J.); deshui_yu@mail.nankai.edu.cn (D.Y.); siyu_li@mail.nankai.edu.cn (S.L.); 5 Institute of Statistics, Nankai University, Tianjin 300071, China; 6 School of Mathematical Sciences, Nankai University, Tianjin 300071, China; jsruan@nankai.edu.cn

**Keywords:** palindromic small RNA, complemented palindromic small RNA, small RNA, DNA complemented palindrome, severe acute respiratory syndrome coronavirus

## Abstract

In this study, we report for the first time the existence of complemented palindromic small RNAs (cpsRNAs) and propose that cpsRNAs and palindromic small RNAs (psRNAs) constitute a novel class of small RNAs. The first discovered 19-nt cpsRNA UUAACAAGCUUGUUAAAGA, named SARS-CoV-cpsR-19, was detected from a 22-bp DNA complemented palindrome TCTTTAACAAGCTTGTTAAAGA in the severe acute respiratory syndrome coronavirus (SARS-CoV) genome. The phylogenetic analysis supported that this DNA complemented palindrome originated from bat betacoronavirus. The results of RNA interference (RNAi) experiments showed that one 19-nt segment corresponding to SARS-CoV-cpsR-19 significantly induced cell apoptosis. Using this joint analysis of the molecular function and phylogeny, our results suggested that SARS-CoV-cpsR-19 could play a role in SARS-CoV infection or pathogenesis. The discovery of cpsRNAs has paved a way to find novel markers for pathogen detection and to reveal the mechanisms underlying infection or pathogenesis from a different point of view. Researchers can use cpsRNAs to study the infection or pathogenesis of pathogenic viruses when these viruses are not available. The discovery of psRNAs and cpsRNAs, as a novel class of small RNAs, also inspire researchers to investigate DNA palindromes and DNA complemented palindromes with lengths of psRNAs and cpsRNAs in viral genomes.

## 1. Introduction

Small RNA sequencing (small RNA-seq or sRNA-seq) is used to obtain thousands of short RNA sequences with lengths that are usually less than 50 bp. With sRNA-seq, many novel non-coding RNAs (ncRNAs) have been discovered. For example, two featured series of ribosomal RNA (rRNA)-derived RNA fragments (rRFs) constitute a novel class of small RNAs [1]. Small RNA-seq has also been used for virus detection in plants [2,3,4] and invertebrates [5]. In 2016, Wang et al. first used sRNA-seq data from the National Center for Biology Information Sequence Read Archive database (NCBI SRA) to show that sRNA-seq can be used to detect and identify human viruses [6], however the detection results were not as robust as those of plant or invertebrate viruses. To improve virus detection in mammals, our strategy was to detect and compare featured RNA fragments in plants, invertebrates, and mammals using sRNA-seq data. In a previous study [7], we detected siRNA duplexes that were induced by plant viruses and analyzed these small interfering RNA (siRNA) duplexes as an important class of featured RNA fragments. In this study, we aimed to investigate siRNA duplexes that were induced by invertebrate and mammalian viruses and unexpectedly discovered another important class of featured RNA fragments: complemented palindromic small RNAs (cpsRNAs). Among all of the detected cpsRNAs, the first discovered cpsRNA, named SARS-CoV-cpsR-19, from the severe acute respiratory syndrome coronavirus (SARS-CoV) strain MA15 merited further study, because mice infected with SARS-CoV MA15 died from an overwhelming viral infection with viral-mediated destruction of pneumocytes and ciliated epithelial cells [8]. SARS-CoV-cpsR-19 was detected from a DNA complemented palindrome TCTTTAACAAGCTTGTTAAAGA in the SARS-CoV genome. In our previous study of mitochondrial genomes, we reported palindromic small RNAs (psRNAs) for the first time [9]. Both of psRNAs and cpsRNAs could comprise a novel class of small RNAs due to their special sequence structures.

In this study, we compared the features of the siRNA duplexes that were induced by mammalian viruses with those that were induced by plant and invertebrate viruses. We found that the detected siRNA duplexes that were induced by mammalian viruses had significantly lower percentages of total sequenced reads than those that were induced by plant and invertebrate viruses, and it seemed that they were produced only from a few sites in the viral genomes. One possible reason could be that a large proportion of the sRNA-seq data is from other small RNA fragments, owing to the presence of a number of double-stranded RNA (dsRNA)-triggered nonspecific responses, such as type I interferon (IFN) synthesis [10]. Another possible reason could be that the missing siRNA duplexes or siRNA fragments function in cells by interacting with host RNAs or proteins. Based on this idea, we hypothesized that SARS-CoV-cpsR-19 or other cpsRNAs from SARS-CoV MA15 could play a role in SARS-CoV infection or pathogenesis.

To test our hypothesis, we conducted the joint analysis of the molecular function and phylogeny. First, we investigated the origins of SARS-CoV-cpsR-19 by the phylogenetic analysis of coronavirus genome sequences that were associated with bats, palm civets, rats, mice, monkeys, dogs, bovines, hedgehogs, giraffes, waterbucks, and equines. Subsequently, we performed RNA interference (RNAi) experiments to test the possible cellular effects that were induced by SARS-CoV-cpsR-19. The phylogenetic analysis supported that the DNA complemented palindrome TCTTTAACAAGCTTGTTAAAGA could originate from bat betacoronaviruses. The results of the RNAi experiments showed that one 19-nt segment corresponding to SARS-CoV-cpsR-19 significantly induced cell apoptosis. This study aimed to provide a different point of view for pathogen detection and pathogenesis studies.

## 2. Materials and Methods

### 2.1. Datasets and Data Analysis

All sRNA-seq data were downloaded from the NCBI SRA database. In our previous study, 6 mammalian viruses (HPV-18, HBV, HCV, HIV-1, SMRV, and EBV) were detected from 36 runs of sRNA-seq data [6]. In this study, 11 invertebrate viruses were detected from 51 runs of sRNA-seq data (Supplementary file 1) and 2 mammalian viruses (H1N1 and SARS-CoV) were detected from 20 runs of sRNA-seq data (NCBI SRA: SRP012018) [11]. In total, 11 invertebrate viruses and 8 mammalian viruses were detected from 107 runs of sRNA-seq data using VirusDetect [4], and their genome sequences were downloaded from the NCBI GenBank database. Among 107 runs of sRNA-seq data, four runs (NCBI SRA: SRR452404, SRR452406, SRR452408, and SRR452410) had been sequenced from lung tissue in mice that were infected with SARS-CoV MA15 [11] and they were used to detect SARS-CoV. The cleaning and quality control of the sRNA-seq data were performed using pipeline Fastq_clean [12], which was optimized to clean the raw reads from the Illumina platforms. Using the software Bowtie v0.12.7 [13] with one mismatch, we aligned all of the cleaned sRNA-seq reads to viral genome sequences and obtained alignment results in sequence alignment map (SAM) format for the detection of siRNA duplexes using the program duplexfinder [7]. Statistical computation and plotting were performed using the software R v2.15.3 (R Core Team, Vienna, Austria) with the Bioconductor packages [14]. The *ORF3b* gene from human betacoronavirus (GenBank: DQ497008.1), 20 homologous sequences from the bat betacoronaviruses, and nine homologous sequences from the civet betacoronaviruses (Supplementary file 1) were aligned using ClustalW2 [15] with curation. After the removal of the identical sequences, the *ORF3b* gene from human betacoronavirus, eight homologous sequences from bat betacoronaviruses, and two homologous sequences from civet betacoronaviruses were used for phylogenetic analysis. Since these homologous sequences had high identities (from 85.16% to 99.78%) to the *ORF3b* gene from DQ497008, the Neighbor Joining (NJ) method was used for phylogenetic analysis.

### 2.2. RNAi and Cellular Experiments

Based on the short hairpin RNA (shRNA) design protocol [1], the 16-nt, 18-nt, 19-nt, 20-nt, and 22-nt segments from the DNA complemented palindrome TCTTTAACAAGCTTGTTAAAGA and their control “CGTACGCGGAATACTTCGA” were selected as the target sequences for pSIREN-RetroQ vector construction (Clontech, Mountain View, CA, USA). PC-9 cells were provided by Dr. Qingsong Wang from Tianjin Medical University and they were divided into six groups—namely, 16, 18, 19, 20, 22, and control—for transfection using plasmids containing the 16-nt, 18-nt, 19-nt, 20-nt, and 22-nt segments and the control sequences. Each group had three replicate samples for plasmid transfection and cell apoptosis measurement. Each sample was processed following the procedure described below. The PC-9 cells were washed with phosphate buffer saline (PBS) and were trypsinized 12 h prior to transfection. Gbico RPMI-1640 medium (Thermo Fisher Scientific, Wattham, MA, USA) was added to the cells, which were then centrifuged at 1000 rpm for 10 min at 4 °C to remove the supernatant. Gbico RPMI-1640 medium containing 10% fetal bovine serum was added to adjust the solvent to reach a volume of 2 μL and contain 2 × 10^5^ cells. These cells were seeded into one well of a 6-well plate for plasmid transfection. Transfection of 2 μg of plasmid was performed using 5 μL Lipofectamine 2000 (Life technology, Carlsbad, CA, USA), following the manufacturer’s instructions. Cell apoptosis was measured with the FITC Annexin V Apoptosis Detection Kit I (BD Biosciences, Franklin Lakes, NJ, USA), following the procedure described below. The cells were washed with PBS, were trypsinized, and were collected using a 5-mL culture tube 48 or 72 h after transfection. The culture tube was then centrifuged at 1000 rpm for 10 min at 4 °C to remove the supernatant. The cells were washed twice with cold PBS and were resuspended in 1X Binding Buffer at a concentration of 1 × 10^6^ cells/mL. Then, 100 μL of the solution (1 × 10^5^ cells) was transferred to a new culture tube with 5 μL of FITC-Annexin V and 5 μL PI. The cells were gently vortexed and were incubated for 15 min at room temperature in the dark. 1X Binding Buffer (400 μL) was added to the tube. Finally, the sample was analyzed using a FACSCalibur flow cytometer (BD Biosciences, Franklin Lakes, NJ, USA) within 1 h. Apoptotic cells were quantified by summing the count of the early apoptotic cells (FITC-Annexin V+/PI−) and the late apoptotic cells (FITC-Annexin V+/PI+). To confirm the results using Annexin V/PI staining and detection, the expression levels of three cell-apoptosis marker genes (*BAX*, *BCL2*, and *CASP3*) were also measured by quantitatice PCR (qPCR) assays using 16-nt, 18-nt, 19-nt, 20-nt, and 22-nt segments to produce siRNA duplexes by pSIREN-RetroQ plasmid transfection. The cells were collected 24 h after transfection. For each gene, three RNAi samples and three control samples were tested for relative quantification. After transfection, RNA extraction, complementary DNA (cDNA) synthesis, and cDNA amplification were performed following the same procedure described below. For each sample, total RNA was isolated using RNAiso Plus Reagent (TaKaRa Bio Inc., Kusatsu, Shiga, Japan) and the cDNA was synthesized by M-MuLV (New England Biolabs, Hitchin, UK). The cDNA product was amplified by qPCR (Eppendorf, Hamburg, Germany) using *GAPDH* as internal control and the qPCR reaction mixture using Real Time PCR Easy (Foregene, Chengdu, China) was incubated at 95 °C for 10 min, followed by 35 PCR cycles (10 s at 95 °C, 20 s at 55 °C, and 30 s at 72 °C for each cycle). The primers of five genes are listed in Table 1.

## 3. Results

### 3.1. Comparison of siRNA Duplexes Induced by Plant, Invertebrate, and Mammalian Viruses

**Figure 1:**
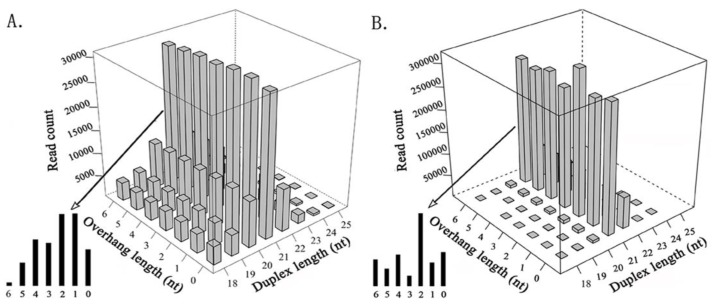
Comparison of small interfering RNA (siRNA) duplexes that were induced by plant and invertebrate viruses. All of the cleaned small RNA sequencing (sRNA-seq) reads were aligned to viral genome sequences using the software Bowtie v0.12.7 [13] with one mismatch. The detection of siRNA duplexes was performed using the program duplexfinder [7]. (**A**). The read count of siRNA duplexes varies with the duplex length and the overhang length, using data from 11 invertebrate viral genomes. (**B**). The read count of siRNA duplexes varies with the duplex length and the overhang length, using data from seven plant viral genomes [7].

In total, 11 invertebrate and 8 mammalian viruses (HPV-18, HBV, HCV, HIV-1, SMRV, and EBV that were detected in the previous study, and H1N1 and SARS-CoV that were detected in this study) were used to detect siRNA duplexes (see Materials and Methods). Next, we compared the features of the siRNA duplexes that were induced by invertebrate viruses (Figure 1A) with those that were induced by plant viruses (Figure 1B). The results showed that duplex length was the principal factor that determined the read count in both plants and invertebrates. The 21-nt siRNA duplexes were the most abundant duplexes in both plants and invertebrates, followed by the 22-nt siRNA duplexes in plants, whereas it was the 20-nt siRNA duplexes in invertebrates. The 21-nt siRNA duplexes with 2-nt overhangs were the most abundant 21-nt duplexes in plants, whereas the 21-nt siRNA duplexes with 1-nt overhangs were the most abundant 21-nt duplexes in invertebrates, however they had a very similar read count to that of the 21-nt siRNA duplexes with 2-nt overhangs. The 18-nt, 19-nt, 20-nt, and 22-nt siRNA duplexes in invertebrates had much higher percentages of total sequenced reads than those in plants. In addition, 18-nt and 19-nt siRNA duplexes had very similar read counts, and the 20-nt and 22-nt siRNA duplexes had very similar read counts in invertebrates. As the siRNA duplexes that were induced by mammalian viruses had significantly lower percentages of total sequenced reads, comparison of the siRNA-duplex features between mammals and invertebrates or plants could not provide meaningful results using our data.

**Figure 2:**
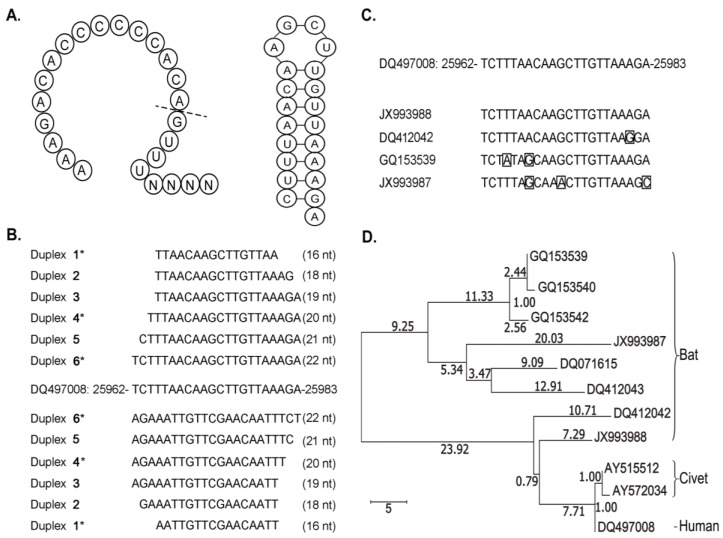
Clues to the origins of SARS-CoV-cpsR-19. All of the genome sequences are represented by their GenBank accession numbers (e.g., DQ497008). (**A**). hsa-tiR-MDL1-16 is derived by cleavage after it starts transcription of the long H-strand primary transcript at the position 561 of human mitochondrial genome (left). SARS-CoV-cpsR-21 can form a hairpin (right). (**B**). 16-nt, 18-nt, 19-nt, 20-nt, and 22-nt siRNA duplexes were used for RNA interference (RNAi) experiments. * 16-nt, 20-nt, and 22-nt palindromic small RNAs (cpsRNAs) were not detected in this study. (**C**). SARS-CoV-cpsR-19 was detected from a DNA complemented palindrome TCTTTAACAAGCTTGTTAAAGA in the SARS-CoV genome. All of the 22-nt homologous sequences in the civet betacoronavirus genomes were identical to the DNA complemented palindrome, whereas four genotypes of 22-nt homologous sequences were detected in the bat betacoronavirus genomes, however only one of them was identical to it. (**D**). The phylogenetic tree was built by the Neighbor Joining (NJ) method using the *ORF3b* gene from human betacoronavirus, eight homologous sequences from bat betacoronaviruses, and two homologous sequences from civet betacoronaviruses. The branch’s length corresponds to an average number of nucleotide changes per 100 nucleotides.

However, as an unexpected result of the comparison, we discovered cpsRNAs from both invertebrate and mammalian viruses. As this study was to focus on the study of cpsRNAs from SARS-CoV, we did not include cpsRNAs from other animal viruses. One 19-nt cpsRNA UUAACAAGCUUGUUAAAGA from a DNA complemented palindrome TCTTTAACAAGCTTGTTAAAGA (DQ497008: 25962-25983), located in the *ORF3b* gene of the SARS-CoV MA15 genome (GenBank: DQ497008.1), was detected in four runs of sRNA-seq data (see Materials and Methods). This 19-nt cpsRNA was named SARS-CoV-cpsR-19 and the DNA complemented palindrome contained 22 nucleotides which perfectly matched its reverse complement sequence. From this DNA complemented palindrome, we also detected one 18-nt and one 21-nt cpsRNA named SARS-CoV-cpsR-18 and SARS-CoV-cpsR-21 (Figure 2A), respectively. however we did not detect cpsRNAs of other lengths (e.g., 16-nt, 20-nt, or 22-nt) using our data. We speculated that 18-nt, 19-nt, and one 21-nt cpsRNA could be derived from siRNA duplexes (Figure 2B), as the presence of these overhanging nucleotides is a hallmark of small RNAs that are produced by silencing-related ribonucleases (e.g., Drosha or Dicer). However, we did not have any evidence to prove the existence of siRNA duplexes.

### 3.2. Discovery of psRNAs and cpsRNAs

Palindromes have been discovered in the published genomes of most species and play important roles in biological processes. The well-known samples of DNA palindromes include restriction enzyme sites, methylation sites, and palindromic motifs in T cell receptors [16]. In this study, we classified DNA palindromes into DNA palindromes and DNA complemented palindromes, from which psRNAs and cpsRNAs could be produced, respectively. A DNA palindrome is classically defined as a nucleic acid sequence that is reverse complementary to itself, while small RNAs that are reverse complementary to themselves are defined as cpsRNAs in this study. Accordingly, a typical psRNA should have a sequence that is 100% identical to its reverse sequence, however most psRNAs are semipalindromic or heteropalindromic, such as hsa-tiR-MDL1-16 AAAGACACCCCCCACA from a DNA palindrome AAAGACACCCCCCACAGTTT (NC_012920: 561-580) [9]. As a heteropalindromic psRNA, hsa-tiR-MDL1-16 is derived by cleavage after it starts transcription of the long heavy strand (H-strand) primary transcript at the position 561 of the human mitochondrial genome (Figure 2A). All cpsRNAs that are discovered from SARS-CoV MA15 are semipalindromic or heteropalindromic, such as SARS-CoV-cpsR-19 and SARS-CoV-cpsR-21. Although SARS-CoV-cpsR-21 contains almost 100% of the total nucleotides which contribute to the matches (Figure 2A), most cpsRNAs have mismatches or insertions/deletions (InDels). One example is a new Epstein-Barr virus (EBV) microRNA precursor (pre-miRNA) with a length of 89 nt, as was reported in our previous study [6]. This pre-miRNA sequence contains only 87.64% (78/89) of the total nucleotides which contribute to the matches.

In this study, we also found that DNA palindromes with sizes ranging from 14 to 31 nt and DNA complemented palindromes with sizes ranging from 14 to 53 nt existed ubiquitously in the animal virus genomes, however only a few of them were detected as transcribed or processed into psRNAs or cpsRNAs. For example, we only detected psRNAs from two (CTACTGACCAGTCATC and AAGGTCTCCCTCTGGAA) of 14 DNA palindromes and cpsRNA from two (GCAAATTGCACAATTTGC and TCTTTAACAAGCTTGTTAAAGA) of 29 DNA complemented palindromes (Table 2) in the SARS-CoV genome (GenBank: DQ497008.1) using four runs of sRNA-seq data (see Materials and Methods). One possible reason could be that only a few of cpsRNAs had been ligated to adapters during the sRNA-seq library preparation process as they could form hairpins (Figure 2A) at room temperature (~20 °C). From Table 2, it can be seen that the T_m_ (melting temperature) of cpsRNA hairpins in the SARS-CoV genome is distributed ranging from 14 °C to 26 °C. This finding provided a clue to explain why the detected siRNA duplexes that were induced by mammalian viruses had significantly lower percentages of total sequenced reads than those that were induced by plant and invertebrate viruses, and could help to improve the virus detection of mammalian viruses.

### 3.3. Clues to Origins of SARS-CoV-cpsR-19

The previously unknown SARS virus generated widespread panic in 2002 and 2003 when it caused 774 deaths and more than 8000 cases of illness. Scientists immediately suspected that civet cats, which are only distant relatives of house cats, may have been the springboard for the transmission of SARS-CoV to humans [17]. Later, scientists concluded that civets were not the original source of SARS. Further investigation showed that the genetic diversity of coronaviruses in bats increased the possibility of variants crossing the species barrier to cause disease outbreaks in the human population [18]. To investigate the origins of SARS-CoV-cpsR-19, we obtained coronavirus genome sequences that were associated with bats, palm civets, rats, mice, monkeys, dogs, bovines, hedgehogs, giraffes, waterbucks, and equines from the NCBI GenBank database. The results of the sequence analysis showed that the DNA complemented palindrome TCTTTAACAAGCTTGTTAAAGA was only located in the *ORF3b* genes of betacoronaviruses. Next, we blasted the *ORF3b* gene of human betacoronavirus (GenBank: DQ497008.1) with all of the obtained betacoronavirus genomes, except for those that were obtained from experiments with mice and monkeys. The results showed that the *ORF3b* gene from human betacoronavirus had homologous genes from the betacoronaviruses of bats and palm civets (Supplementary file 1) rather than from those of other species. The DNA complemented palindrome also had 22-nt homologous sequences in the bat and civet betacoronavirus genomes. All of the 22-nt homologous sequences in the civet betacoronavirus genomes were identical to the DNA complemented palindrome, whereas four genotypes of 22-nt homologous sequences were detected in the bat betacoronavirus genomes, however only one of them was identical to it (Figure 2C). Four genotypes had no, one, two, and three mismatches with the DNA complemented palindrome and their corresponding *ORF3b* homologous sequences had identities of 96.77%, 96.13%, 87.96%, and 85.16%, respectively. This suggested that one betacoronavirus variant containing the DNA complemented palindrome could have originated from bats and was then passed onto palm civets and finally to humans. This was consistent with the results of the phylogenetic analysis using the *ORF3b* homologous sequences from the bat and civet betacoronavirus genomes (Figure 2D). In the phylogenetic tree, all of the human and civet betacoronaviruses containing the DNA complemented palindrome were grouped into one clade. The nearest relative of the human and civet clade was the bat betacoronavirus (GenBank: JX993988.1) containing the DNA complemented palindrome, and the next nearest relative was the bat betacoronavirus (GenBank: DQ412042.1) containing a homologous 22-nt sequence with one mismatch with the DNA complemented palindrome.

### 3.4. Preliminary Studies on Biological Functions of SARS-CoV-cpsR-19

**Figure 3:**
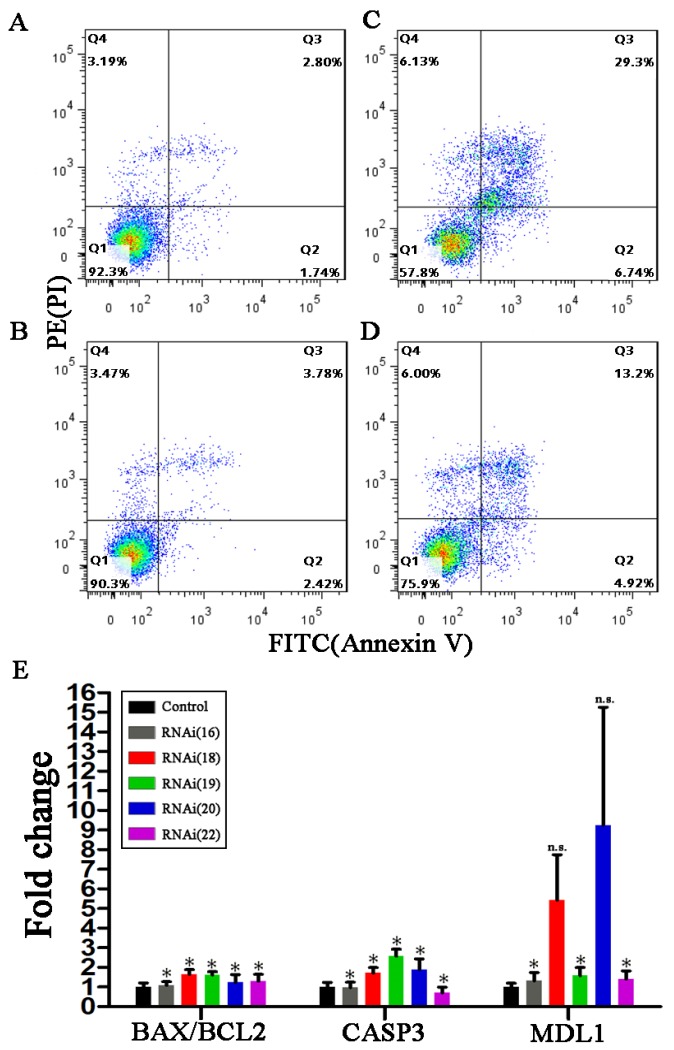
RNAi and cellular experiments for validation. PC-9 cells were divided into six groups named 16, 18, 19, 20, 22, and control for transfection using plasmids containing 16-nt, 18-nt, 19-nt, 20-nt, 22-nt segments, and their controls (Figure 2B). Each group had three replicate samples for plasmid transfection and cell apoptosis measurement. The 19-nt and 20-nt segments significantly induced cell apoptosis, whereas the 16-nt, 18-nt, and 22-nt did not show significantly positive results. The samples in this figure were selected randomly from the control (**A**), 18 (**B**), 19 (**C**), and 20 (**D**) group. (**E**). The experimental results by qPCR assays were consistent with those using Annexin V/PI staining and detection. The primers of *MDL1* were used to amplify both *MDL1* and *MDL1AS*, as they are sense–antisense transcripts from the same loci [9] and are predicted to be markers which can indicate the activities of mitochondria and even the whole cells.

Our previous study showed that the psRNA hsa-tiR-MDL1-16 contained the Transcription Initiation Site (TIS) of the human mitochondrial H-strand and could be involved in mtDNA transcription regulation [9]. This inspired us to speculate that cpsRNAs could also have specific biological functions and we investigated SARS-CoV-cpsR-19 using RNAi and Annexin V/PI staining and detection. 16-nt, 18-nt, 19-nt, 20-nt, and 22-nt segments from the DNA complemented palindrome TCTTTAACAAGCTTGTTAAAGA were used to produce siRNA duplexes by pSIREN-RetroQ plasmid transfection (see Materials and Methods). As a result, the 19-nt and 20-nt segments significantly induced cell apoptosis 2.76- and 1.48-fold 48 h after their transfection, respectively. Particularly, the 19-nt segment significantly induced cell apoptosis 7.94-fold (36.04%/4.54%) 72 h after its transfection into PC-9 cells (Figure 3). Using the 19-nt segment, we also tested cell apoptosis in five other human cell lines and one mouse cell line. The results showed that the 19-nt segment significantly induced cell apoptosis in the A549, MCF-7, and H1299 cell lines, however it did not in the MB231, H520, and L929 (mouse) cell lines. The siRNA duplexes that were induced by the 19-nt segment could silence cell-specific transcripts to induce cell apoptosis through RNAi. These results suggested that SARS-CoV-cpsR-19 had significant biological functions and could play a role in SARS-CoV infection or pathogenesis.

To confirm the results using Annexin V/PI staining and detection, the expression levels of three cell-apoptosis marker genes (*BAX*, *BCL2*, and *CASP3*) were measured by qPCR assays using the same RNAi protocol in the Hela cell line (see Materials and Methods). The results showed 1.08-, 1.65-, 1.60-, 1.24-, and 1.30-fold increases in *BAX/BCL2* ratios which were caused by 16-nt, 18-nt, 19-nt, 20-nt, and 22-nt segments, respectively (Figure 3E). As a low *BAX/BCL2* ratio (<1) and a high *BAX/BCL2* ratio (>1) is against and in favor of cell apoptosis, it suggested that 18-nt and 19-nt segments significantly induced cell apoptosis. The RNAi using 18-nt, 19-nt, and 20-nt segments also caused significantly increased expression of *CASP3* by 1.72, 2.58, and 1.89 fold changes in Hela cells. Therefore, the experimental results by qPCR assays were consistent with those using Annexin V/PI staining and detection. In addition, the expression levels of two novel long non-coding RNAs (lncRNAs) (*MDL1* and *MDL1AS*) from mitochondrial genome were measured to investigate their expression changes that were caused by RNAi using 16-nt, 18-nt, 19-nt, 20-nt, and 22-nt segments. *MDL1* and *MDL1AS* were recently discovered [9] and were predicted to be markers which can indicate the activities of mitochondria and even the whole cells. The RNAi using 16-nt, 18-nt, 19-nt, 20-nt, and 22-nt segments also caused increased relative expression levels of *MDL1* by 1.28, 5.43, 1.58, 9.25, and 1.41 fold changes in Hela cells (Figure 3E). It suggested that qPCR of *MDL1* produce higher sensitivities than that of *BAX/BCL2* and *CASP3* in the detection of cell apoptosis.

## 4. Conclusions and Discussion

SARS-CoV-cpsR-19 from the DNA complemented palindrome TCTTTAACAAGCTTGTTAAAGA (DQ497008: 25962-25983) is located in the *ORF3b* gene of the SARS-CoV MA15 genome. In one previous study, this 22-nt DNA palindrome was studied using two mathematics models (M0 and M1) to conclude that this DNA palindrome was quite unlikely to occur by chance [19]. These authors also found that the underrepresentation of 4-nt DNA palindromes existed in all of the coronaviruses and the significant underrepresentation of 6-nt DNA palindromes existed only in SARS but not in the other six coronaviruses. Then, they hypothesized that this avoidance of 6-nt DNA palindromes in the SARS genome would offer a protective effect on the virus, making it comparatively more difficult to be destroyed by some host-defence mechanisms (e.g., restriction enzymes). In this study, we unexpectedly discovered cpsRNAs from the DNA palindrome TCTTTAACAAGCTTGTTAAAGA and reported that SARS-CoV-cpsR-19 could play a role in SARS-CoV infection or pathogenesis. We hypothesized that cpsRNAs, as a novel class of small RNAs, were produced by some host-defence mechanisms (e.g., RNAi) and that they could interact with the immune system of the host cells to help virus survival or infection.

Another previous study reported that another sRNA AGGAACUGGCCCAGAAGCUUC in the SARS-CoV genome, named small viral RNA-N (svRNA-N), contributed to SARS-CoV pathogenesis [20]. SARS-CoV-cpsR-19 was detected in very low abundance using four runs of sRNA-seq data (see Materials and Methods) in this study but was not detected using the sRNA-seq data (NCBI SRA: SRP094035) in that study [20]. One possible reason could be that SARS-CoV-cpsR-19 can form one hairpin and the T_m_ (melting temperature) of this hairpin is about 20 °C. Therefore, most of SARS-CoV-cpsR-19 cannot be ligated to adapters during the sRNA-seq library preparation process at room temperature (~20 °C). As the optimal secondary structure of svRNA-N (Minimum Free Energy (MFE) = −1.7) is less stable than those of SARS-CoV-cpsR-19 (MFE = −2.6) and all the other possible cpsRNAs (Table 2), svRNA-N is more easily captured during the sRNA-seq library preparation process. SARS-CoV-cpsR-19 induced cell apoptosis, whereas svRNA-N caused pulmonary inflammation. In Vivo experiments revealed that the biogenesis pathway that was responsible for svRNA-N’s synthesis was not dependent on Dicer or Drosha as the canonical biogenesis of miRNAs and svRNA-N were able to silence mRNA expression by targeting its 3’ UTR. However, the mechanisms underlying infection or pathogenesis of both SARS-CoV-cpsR-19 and svRNA-N are still unknown.

## Figures and Tables

**Table 1:** Primers for quantitative PCR (qPCR) assays.

Gene Symbol	Forward Primer	Reverse Primer
*GAPDH*	ACATCGCTCAGACACCATG	TGTAGTTGAGGTCAATGAAGGG
*BAX*	AGTAACATGGAGCTGCAGAG	AGTAGAAAAGGGCGACAACC
*BCL2*	GTGGATGACTGAGTACCTGAAC	GCCAGGAGAAATCAAACAGAGG
*CASP3*	ACTGGACTGTGGCATTGAG	GAGCCATCCTTTGAATTTCGC
*MDL1* *	CCCAATCCACATCAAAACCC	GGACGAGAAGGGATTTGACTG

* The primers of *MDL1* were used to amplify both *MDL1* and *MDL1AS* as they are sense–antisense transcripts from the same loci [9] and were predicted to be markers which can indicate the activities of mitochondria and even the whole cells.

**Table 2:** DNA complemented palindromes in the severe acute respiratory syndrome coronavirus (SARS-CoV) genome.

DNA Complemented Palindrome	Start	End	Length	GC%	Tm	MFE
GGTAACTATAAAGTTACC	1783	1800	18	33	20	−6.9
AATGTGAGAATCACATT	2779	2795	17	29	18	−4.4
AAGAAACTAAGTTTCTT	3923	3939	17	24	18	−3.5
ATGGTAAGCTTTACCAT	3971	3987	17	35	18	−4.5
AAATGCAAATCTGCATTT	4234	4251	18	28	18	−4.9
ATATGTCTATGACATAT	4949	4965	17	24	18	−4.3
CCTCATGTAAATCATGAGG	5020	5038	19	42	22	−9.0
ATAACAATTGTTAT	5207	5220	14	14	12	−0.9
ACTTCAACAGCTTGAAGT	5241	5258	18	39	18	−4.7
ACTTCAAATTCATTTGAAGT	6256	6275	20	25	20	−5.5
GTACTTTTACTAAAAGTAC	6734	6752	19	26	20	−4.6
ATCTACCAGTGGTAGAT	9189	9205	17	41	20	−6.5
TTACCTTCCAAGGTAA	10,892	10,907	16	38	16	−4.0
CCACTTATTAAGTGG	14,160	14,174	15	40	18	−4.7
CCCATTTAATAAATGGG	14,882	14,898	17	35	20	−5.7
CAGTGACAATGTCACTG	16,463	16,479	17	47	22	−7.7
CACCTTTGAAAAAGGTG	16,760	16,776	17	41	20	−5.6
TGTAAGAGAATTTCTTACA	17,651	17,669	19	26	18	−5.3
TGAATATGACTATGTCATATTCA	17,783	17,805	23	26	26	−9.7
CTACTTTAAGAAAGTAG	20,081	20,097	17	29	18	−3.6
AGCATTCTTGGAATGCT	21,106	21,122	17	41	20	−6.1
TTCCTCTTAAATTAAGAGGAA	21,337	21,357	21	29	22	−9.1
GCATTACTACAGAAGTAATGC	23,599	23,619	21	38	22	−7.9
AGCCCTTTATAAGGGCT	25,480	25,496	17	47	22	−8.7
TCTTTAACAAGCTTGTTAAAGA *	25,962	25,983	22	27	20	−7.2
CAACGGTACTATTACCGTTG	26,406	26,425	20	45	24	−9.0
ACCTTCATGAAGGT	28,048	28,061	14	43	14	−2.6
GCAAATTGCACAATTTGC *	29,028	29,045	18	39	18	−4.0
TAAAATTAATTTTA	29,668	29,681	14	0	NA	NA

In total, 29 DNA complemented palindromes were identified in the SARS-CoV genome (GenBank: DQ497008.1). * In this study, we only detected psRNAs from two of 29 DNA complemented palindromes. Minimum Free Energy (MFE) was calculated using RNAfold (http://rna.tbi.univie.ac.at/cgi-bin/RNAWebSuite/RNAfold.cgi). Tm (melting temperature) of cpsRNA hairpins was calculated by the formula: Tm = 4*(C+G) + 2*(A+T) and only using the nucleic acids in the stems of DNA complemented palindromes.

## Supplementary Materials

The following are available online at http://www.mdpi.com/2073-4425/9/9/442/s1, Table S1: 51 runs of sRNA-seq data for invertebrate virus detection, Table S2: Homologous sequences of ORF3b.
